# Intrinsic Brain Network Biomarkers of Antidepressant Response: a Review

**DOI:** 10.1007/s11920-019-1072-6

**Published:** 2019-08-13

**Authors:** Katharine Dunlop, Aleksandr Talishinsky, Conor Liston

**Affiliations:** 1 Brain and Mind Research Institute, Weill Cornell Medicine, 413 East 69th Street, Box 240, New York, NY 10021 USA; 2 Department of Psychiatry, Weill Cornell Medicine, New York, NY USA

**Keywords:** Major depressive disorder, Biomarker, Treatment response, Predictor, Brain networks, Network connectivity

## Abstract

**Purpose of Review:**

Poor treatment response is a hallmark of major depressive disorder. To tackle this problem, recent neuroimaging studies have sought to characterize antidepressant response in terms of pretreatment differences in intrinsic functional brain networks. Our aim is to review recent studies that predict antidepressant response using intrinsic network connectivity. We discuss current methodological limitations and directions for future antidepressant biomarker studies.

**Recent Findings:**

Functional connectivity stemming from the subgenual and rostral anterior cingulate has shown particular consistency in predicting antidepressant response. Differences in this connectivity may prove fruitful in differentiating treatment responders to many antidepressant interventions. Future biomarker studies should integrate biological MDD subtypes to address the disorder’s inherent clinical heterogeneity.

**Summary:**

These clinical and scientific advancements have the potential to address this population marked by limited treatment response. Methodological considerations, including patient selection, response criteria, and model overfitting, will require future investigation to ensure that biomarkers generalize for prospective prediction of treatment response.

## Introduction

A hallmark feature of major depressive disorder (MDD) is patients’ limited treatment responsivity to conventional pharmacotherapy and psychotherapy. As demonstrated by the seminal STAR*D trial, approximately one third of patients remit to their first antidepressant, and a given patient’s likelihood of remission diminishes with each successive monotherapy [1]. Those who do not respond to two or more antidepressant trials are considered to have treatment-resistant depression (TRD); TRD is estimated to affect 35% of MDD patients [2]. These limited response rates may be related to the fact that MDD is a highly heterogeneous disorder [3••], and individual patients may subsequently require tailored treatments.

Motivated by these poor response rates, efforts have been made to identify pretreatment clinical characteristics that predict response to antidepressant interventions [4]. For example, comorbid psychiatric or somatic disorders are associated with poorer outcomes to pharmacotherapies. In the STAR*D trial, the remission rate of patients with severe somatic symptoms dropped to only 29.3% across three successive monotherapies [5]. It is important to note that although clinical factors such as somatic symptoms show significance at the group level, none have yet achieved the much higher bar of clinically meaningful predictive value at the individual level.

In light of these studies showing limited individual predictive value for clinical measures, recent studies have sought to advance our understanding of antidepressant response in terms of pretreatment differences in biological measures. Abnormal structural and functional connectivity within or between functional brain networks are present across a wide variety of psychiatric illnesses [6]. Promising results have been reported in a growing oeuvre of neuroimaging studies that have assessed the accuracy of MRI connectivity measures in predicting antidepressant response [7].

In this article, our aim is to review recent studies identifying intrinsic brain network differences that predict antidepressant response. First, we provide a brief background on functional brain networks and the specific networks pertinent to MDD. Next, we review recent studies reporting pretreatment differences in brain network activity or connectivity that characterize treatment response. Finally, we discuss current clinical challenges and methodological drawbacks, and comment on areas of improvement for the next generation of antidepressant biomarker studies.

## Intrinsic Brain Networks and Their Relationship to MDD

### What Are Intrinsic Brain Networks?

Over the past two decades, functional neuroimaging studies have consistently found that the brain exhibits spontaneous, low-frequency fluctuations in the fMRI BOLD signal and that interregional correlations in these fluctuations can be used to define large-scale networks that are anatomically and functionally distinct [8]. These networks are discernible from both ongoing spontaneous fluctuations in brain activity [9] and from neuroimaging harnessing cognitive or behavioral paradigms [10]. Consequently, we term these networks intrinsic brain networks (IBNs): distinct networks of functionally coupled brain regions whose spontaneous or task-evoked fluctuations in activity are correlated over time [9]. The spatial motifs of IBNs are highly replicable, with strong intraindividual [11] and interindividual consistency [12].

Large-scale IBNs are also hypothesized to reflect the underlying structural topology of the brain [13, 14], and connectivity within or between IBNs has been associated with human cognition and behavior through a variety of paradigms, including cognitive control tasks [15], motor and language performance [16], impulsivity [17], and states of consciousness [18]. While not yet fully understood, current studies suggest that IBNs operate in concert to facilitate complex cognitive and behavioral processes [19].

### Functional Networks Pertinent to MDD Symptomatology

**Fig. 1:**
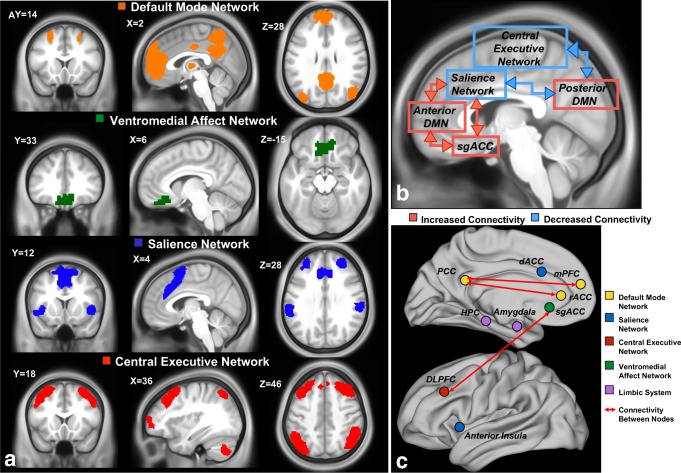
Intrinsic brain networks may be used to differentiate depressed treatment responders from nonresponders. **a** The four intrinsic brain networks implicated in MDD (default mode = yellow, salience = blue, central executive = red, and ventromedial reward = green). Networks were generated using [23]*.* **b** Connectivity changes commonly reproduced in depressed patients compared to healthy controls (adapted from [24]). Red boxes and lines indicate increased within-network and between-network connectivity, respectively. Blue boxes and lines indicate decreased within-network and between-network connectivity, respectively. **c** Nodes and connectivity implicated in predicting antidepressant treatment response, colored by intrinsic brain network membership

While the exact number of IBNs is not yet fully known, most human neuroimaging studies report the existence of 7–17 IBNs [19, 20]. Furthermore, studies of structural and functional MRI, electroencephalography, and positron emission tomography have identified at least four candidate IBNs involved in MDD pathophysiology: the default mode network (DMN), salience network (SN), central executive network (CEN), ventromedial affect network (VMN), and IBNs related to autonomic and limbic function (reviewed extensively in [21, 22]) (Fig. 1a, b). To summarize, negative self-referential rumination is associated with disrupted connectivity of the SN and CEN with the DMN and VMN, as well as hyperactivation stemming from the subgenual cingulate cortex [25, 26]. Similarly, MDD patients exhibit DMN and VMN hyperactivity to aversive or nonrewarding stimuli [27–29]. Conversely, hypoactivity of attentional and ventromedial reward networks (SN and VMN) has been linked to poor incentive salience and anhedonia, another hallmark MDD symptom [30–32]. MDD patients also display deficits in cognitive control compared to nondepressed controls, and this deficiency has been attributed to abnormal functioning of the SN and CEN [33–36].

## Brain Network Biomarkers of Antidepressant Response

### Default Mode Network

The DMN is comprised of the posterior cingulate cortex (PCC), precuneus, medial prefrontal cortex (mPFC), rostral anterior cingulate cortex (rACC), bilateral inferior parietal cortex (IPL), and medial/lateral temporal cortices [37] (Fig. 1a). Recent studies have reported that DMN activation is related to a number of behaviors related to internally generated cognition and self-referential processing [38], including mind wandering [39], autobiographical memory retrieval [40], spatial navigation, and theory of mind [41]. Furthermore, the DMN appears to consist of at least three subnetworks: a midline “core” network comprised of the mPFC and PCC that is consistently activated for all DMN-relevant functions; a dorsal mPFC subnetwork that co-activates with the angular gyrus and temporal pole for self-referential or affective processes; and a temporoparietal network comprised of the IPL, temporal cortices for memory retrieval and scene reconstruction [20, 42].

Recent studies frequently report that volumetric decreases and functional activity of the midline “core” subnetwork predict antidepressant response. Anteriorly, larger baseline rACC/mPFC volume is significantly associated with response to fluoxetine [43], chronotherapeutics [44], and internet-based cognitive therapy [45]. In older adults, rACC volume [46] and white mater integrity is positively correlated with escitalopram response and improvements in negative self-referential rumination [47]. However, pretreatment rACC volume is not significantly associated with response to all pharmacotherapy. For sertraline response, rACC volume increases within the first week of treatment and is significantly correlated with improvement at 8 weeks; however, these structural indices individually predicted escitalopram response with an accuracy of only 65% [48•]. Functionally, resting-state rACC theta has been correlated with antidepressant response in two large pharmacotherapy trials with conflicting results: one study reported that high frontal theta was associated with nonresponse [49], while the other showed the converse [50••].

Posterior nodes of the midline subnetwork have also been shown to correlate with antidepressant response. For example, larger baseline PCC volume is correlated with pharmacotherapy response [51], and higher pretreatment glucose metabolism in the precuneus is linked to psychotherapy completion [52]. Additionally, deactivation of both the precuneus and PCC during an emotion discrimination task correlated with improvements after 2 weeks of pharmacotherapy, but not after 4 weeks [53].

Connectivity between the anterior and posterior regions of the DMN also correlates with antidepressant response. Five recent studies have reported that stronger baseline posterior DMN (PCC) connectivity to the anterior DMN (mPFC) correlates with antidepressant response to electroconvulsive therapy (ECT) [54] and pharmacotherapy [55–58]. Furthermore, four of these studies were able to correctly classify participants as responders with > 80% accuracy [54, 55, 57, 58].

### Ventromedial Affect and Reward Networks

Affective and reward networks have long been implicated in the pathophysiology of depression: the subgenual anterior cingulate cortex (sgACC), a central node involved in affect, was one of the earliest neuroimaging biomarkers of depression and antidepressant response [28, 59–63]. Since then, an affect-associated network with strong connectivity to the sgACC has been repeatedly implicated in depressive affect and treatment response [30, 64]. In this review, we broadly define the VMN as encompassing the sgACC, nucleus accumbens (NAcc), medial OFC (mOFC), and ventromedial prefrontal cortex (VMPFC) [65] (Fig. 1a).

Within the past decade, neuroimaging has provided several lines of evidence supporting a role of the sgACC in predicting antidepressant response. Importantly, variations in sgACC structure, function, and connectivity have been associated with response to many different interventions. These variations suggest that sgACC function may be used to guide treatment selection. For example, early PET studies reported that treatment response can be associated with both increased and decreased sgACC metabolism for noninvasive brain stimulation [66] and CBT combined with SSRI treatment, respectively [67, 68]. In addition to baseline metabolic activity, structural MRI studies have shown that greater baseline sgACC volume correlated with clinical response to CBT [69] and ECT [70]. Some [71, 72] but not all [73] task-based fMRI studies have supported the notion that lower sgACC activity in response to emotional stimuli predicts response to CBT.

Similar to studies assessing structural, metabolic, and task-evoked biomarkers, resting-state fMRI studies have shown sgACC functional connectivity to predict treatment response to pharmacotherapy, psychotherapy, and TMS. Crucially, the spatial topography of sgACC functional connectivity may help to guide treatment selection. Studies investigating connectivity between sgACC and prefrontal regions, including the dorsomedial prefrontal cortex (DMPFC), dorsolateral prefrontal cortex (DLPFC), VMPFC, ventrolateral prefrontal cortex (VLPFC), and medial orbitofrontal cortex, have shown more positive baseline functional connectivity to be associated with improved treatment outcome for pharmacotherapy and psychotherapy [74, 75••, 76–78]. On the other hand, more negative baseline functional connectivity between sgACC and the ACC, DMN, and parts of the left superior mPFC has also been associated with improved treatment outcome for noninvasive brain stimulation targeting the DLPFC [58, 78].

### Salience and Attentional Networks

The SN is predominantly comprised of the dACC and anterior insula (AI) [79] (Fig. 1a), and its activity is related to the detection of and autonomic reactions to salient stimuli [80]. SN activity is related to goal-directed behavior, meaning that the SN identifies relevant external information [81, 82] and projects to motor and premotor regions via the dACC to initiate a behavior [83, 84]. AI and dACC activity is also positively correlated with affective face discrimination [85].

Four studies have reported that antidepressant response is associated with pretreatment dACC activity during emotion processing and affect regulation. Escitalopram response was associated with high baseline dACC activity to faces displaying negative versus positive affect [86], with a greater reduction in this contrast after the first week of treatment [87]. However, baseline dACC hyperactivity correctly classified escitalopram response at roughly 70% accuracy [86]. Baseline dACC hyperactivity during emotion processing has also significantly correlated to antidepressant response to chronotherapeutics [44] and venlafaxine [88] with comparable classification accuracy.

Insula activity during emotion processing has also been linked with antidepressant response, with hyperactivity during negative emotional images being predictive of response to mirtazapine, venlafaxine [89], and combination pharmacotherapy [90]. Similarly, a greater reduction in insular activity to negative facial expressions within the first week of escitalopram is predictive of later antidepressant response [87]. Also of note, insula hyper- or hypometabolism may discriminate between response to pharmacotherapy versus psychotherapy, with hyperactivity corresponding to escitalopram response and the opposite to cognitive behavioral therapy [91].

Notably, SN activity has been shown to predict antidepressant response in TRD to repetitive transcranial magnetic stimulation (rTMS) targeting both the DLPFC and DMPFC. DLPFC-rTMS responders display increased SN functional connectivity on resting-state fMRI [92] and theta power and connectivity using electroencephalography (EEG) [93]. Both of these studies report sensitivities and specificities > 80% using these measures. DMPFC-rTMS responders display higher baseline SN activity to the sgACC in one early open-label study [74], highlighting the potential of noninvasive neurostimulatory targets to access networks that are modulated by invasive therapeutics like DBS [94].

### Frontoparietal Central Executive Network

The DLPFC, frontal eye fields, and superior parietal cortex are the core nodes of the CEN. The CEN supports the identification of changes in the environment that necessitate the inhibition or adaptation of behaviors [95], and CEN activity is implicated in a diverse array of behaviors that includes action planning, sustaining attention, working memory, behavioral inhibition, and cognitive flexibility [96].

DLPFC activity during working memory and cognitive control tasks appears to be a candidate biomarker of pharmacotherapy response. Baseline DLPFC activation during response inhibition that resembled activation in healthy controls differentiated remitters from nonremitters to sertraline, escitalopram, and venlafaxine [97•]. Furthermore, greater DLPFC activity during working memory task was correlated with antidepressant response, but only in patients without childhood maltreatment [98]. Also from the same trial, frontoparietal CEN volume predicted nonremission in a subset of patients [99].

Notably, the DLPFC is also an established neurostimulation target using rTMS, and consequently, its functional correlates have been associated with response to rTMS. High frontostriatal connectivity from the DLPFC [100] has been associated with favorable response to left DLPFC-rTMS. Similar to studies highlighting the relationship between DBS and rTMS targets [74, 94], low baseline connectivity between the DLPFC and sgACC [101••] has also been shown to correlate with antidepressant response to DLPFC-rTMS. Furthermore, electrophysiological DLPFC dynamics, including alpha power [102] and entropy [103], have also been linked to antidepressant response to rTMS.

### Limbic and Autonomic Circuitry

Early neuroimaging studies of the limbic system found associations between depression and changes in limbic structures, including decreased hippocampal volume [104], decreased amygdala volume [105], and increased amygdala glucose metabolism [106]. These same changes have also been associated with treatment response; increased hippocampal volume was shown to correlate with improved response to antidepressants [104], increased amygdala volume predicts response to ECT [107] and ketamine [108], and decreased amygdala glucose metabolism correlates with improved response to paroxetine [109]. Overall, limbic structural markers may be seen as an indicator of depression severity, with less severe structural abnormality associated with improved treatment response.

Connectivity between limbic and prefrontal structures has also been shown to be abnormal in MDD in the context of emotional regulation, leading to the hypothesis that decreased top-down control of limbic brain regions by prefrontal input serves as a neural substrate of depressive affect [110]. Recently, fMRI studies have revealed that abnormalities in corticolimbic connectivity in depressed patients are predictive of antidepressant response. Specifically, improved antidepressant response has been correlated with more positive amygdala–vmPFC connectivity [58], more positive amygdala to VLPFC and to ACC connectivity [111], higher amygdala connectivity with right central operculum [112], and lower connectivity with the supplementary motor area and precentral gyrus [112]. These studies support the hypothesis that stronger top-down control of amygdala activity by prefrontal cortices can predict improved treatment outcome.

### Internetwork Connectivity as Antidepressant Biomarkers

It is important to note that the biomarkers reported by many studies do not perfectly align with one IBN, but rather report that connectivity of nodes between many networks is predictive of antidepressant response. For example, tractography studies have reported that sgACC-DBS response correlates with electrode locations that stimulate a distributed network consisting of the amygdala, VMPFC, rACC, dACC, and NAc [113•, 114]. More recently, baseline network characteristics or MDD subtypes based on patterns of whole-brain connectivity have shown promise in predicting antidepressant response to rTMS. We recently showed that functional connectivity-based subtypes of MDD related to anhedonia and anxiety could be used to predict response rates to DMPFC-rTMS [3••]. Furthermore, whole-brain clustering and network efficiency has been shown to correlate with antidepressant response to accelerated DLPFC-rTMS [115].

### Summary

Baseline differences localized to structural and functional connectivity of the sgACC, DMN, SN, DLPFC, hippocampus, and amygdala predict treatment response (Fig. 1c). However, there does not seem to be a “one-size-fits-all” biomarker for MDD interventions. More specifically, no single biomarker predicts response for all antidepressant interventions or patient populations. Rather, the combined use of complementary biomarkers may allow the comparison of predicted response to different treatments, thereby guiding treatment selection.

At least in adult MDD populations, the most consistently replicated biological marker of antidepressant response is connectivity stemming from the subgenual and rostral ACC. In particular, divergent sgACC connectivity appears to be predictive of antidepressant response to a variety of interventions. sgACC connectivity to nodes of the VMN, SN, and CEN—the ventrolateral PFC, insula midbrain, and striatum—differentially predicts response to CBT and pharmacotherapy [75••]. In contrast, connectivity to the ACC and DLPFC—nodes of the SN and CEN, respectively—is predictive of response to rTMS [74, 92, 101••]. Furthermore, structural connectivity from the sgACC to the striatum, ventral PFC, and dorsal PFC is implicated in response to sgACC-DBS [113•].

The mPFC/rACC node of the DMN is also implicated in many biomarker studies, including those involving pharmacotherapy [50••], placebo [116], and rTMS [92]. Two large studies from the same consortium report that intact activity of the rostral ACC relative to healthy controls—lower theta power and higher resting-state functional connectivity to the DMN—predicted nonspecific response to three different pharmacotherapies [49, 57]. However, many other studies, albeit without a healthy comparator group, report that higher rACC theta and connectivity to the SN is predictive of nonspecific antidepressant response, including response to placebo [48•, 50••, 116]. Further work is needed to elucidate the relationship between IBN biomarkers of response in comparison to a healthy comparator group.

MDD is also a clinically heterogeneous disorder, and so it is likely that biomarkers attributed to specific MDD subtypes may be required to tailor treatment selection and improve prediction accuracy. To date, one study has identified biological subtypes of MDD that differentially respond to dmPFC-rTMS [3••]. In this study, individuals who possessed resting-state functional connectivity abnormalities related to anxiety and insomnia symptom severity responded to rTMS, integrating biologically based MDD subtypes, clinical dimensions, and functional MRI [3••]. Future studies should be mindful of the clinical heterogeneity of MDD when identifying biological predictors of response.

## Methodological Challenges to Identifying MDD Biomarkers

### Patient Selection

One area of methodological variability is enrollment criteria. While most studies enrolled adults in a current major depressive episode using DSM-IV or DSM-5 criteria, many identify IBN biomarkers in patients diagnosed with late-life depression [46], adolescent MDD [112], bipolar disorder in a current depressive episode [44], TRD [74], or MDD presenting with other comorbidities [58]. The neurobiological correlates of demographic, diagnostic, and symptom heterogeneity in MDD are not yet fully understood. Consequently, it is possible that the biomarkers generated with modest sample sizes of clinically heterogeneous populations will be challenging to independently replicate, unless investigators take care to replicate the recruitment conditions and diagnostic sample, in addition to the other methods. Identifying biological subtypes based on common abnormal brain function and symptomatology in large trials has been a next logical next step, with our group recently demonstrating that stratifying a heterogeneous group of patients into biologically based subtypes significantly improves prediction accuracy of response to rTMS [3••]. Other examples of using fMRI measures of functional connectivity to parse diagnostic heterogeneity in depression and related affective disorders have also yielded promising results [117–119].

### How Do We Define Treatment Response?

Another critical area of heterogeneity is in each study’s definition of treatment response. For example, some studies report linear correlations of neuroimaging measures with the degree of clinical improvement or improvement of a particular symptom. Other biomarkers were generated using categorical predictors of “response,” typically > 50% of a primary outcome measure from baseline to a certain time point into treatment. More challenging, however, is that the therapeutic window that defines “response” for the same intervention varies considerably. For example, escitalopram studies have reported qualitatively different biomarkers based on response at 6 [87], 8 [49], 10 [120], or 12 weeks [68] from intervention onset. Similarly, IBN biomarkers have been identified for CBT response at 12 weeks [68], 14 sessions [73], and 22 sessions [69]. This heterogeneity limits how biomarkers may generalize to independent datasets, and this timeframe need to be clarified in future studies if they are to be translated for wider clinical use. Further longitudinal studies aimed at understanding whether and how the predictors of treatment response at one time point (e.g., 6 weeks) differ from those predicting response at another time point (e.g., 12 weeks).

### Considerations for Individual-Level Prediction

Overfitting is an important consideration in publications that employ machine learning and cross-validation techniques to assess individual-level prediction. Classification accuracy and, therefore, model overfitting is dependent on a number of factors, including the sample size, feature selection, classification methods, and cross-validation [121•]. In one example discussed by Gao and colleagues in their recent review on the machine learning in MDD [121•], leave-one-out cross-validation improves the classification accuracy of a model because more data is provided during training, but can result in poor model performance in new datasets because the performance of the model is highly dependent on the training dataset. It will be important for future studies to account for the possibility of overfitting by employing methods of feature selection, regression, and classification that limit bias and overfitting [122], as well as machine learning and cross-validation methods that are less sensitive to the sample size of the training dataset [121•].

## Moving the Field Forward

Given that less than one third of MDD patients remit on first treatment course [1], prognostic biomarkers of treatment response for specific therapies are in high demand. As outlined in this review, many prognostic biomarkers have been developed for a variety of MDD therapies. However, these neuroimaging biomarkers have yet to be employed in a clinical setting.

A number of practical barriers may prevent neuroimaging biomarkers from being easily measured in the clinical setting, including cost, accessibility, and time to interpretation. For one, many psychiatric clinical settings lack MRI access and on-site expertise [123]. Furthermore, it may not be feasible to schedule, acquire, process, and interpret neuroimaging results, unless these steps can be implemented rapidly. One future possibility might be to collectively concentrate the biomarker search within just one or two MDD subpopulations to minimize the demand on resources while maximizing the potential benefit. TRD biomarkers, for example, might be the most fruitful start, with the aim of identifying the optimal treatment between combination/augmentation pharmacotherapy and more invasive interventions, such as rTMS and ECT, or between different cortical rTMS targets, including the DLPFC and DMPFC.

Moving forward, biomarkers of antidepressant response may be bolstered by the integration of multiple modalities. Recent studies integrating structural and functional neuroimaging have shown that the prediction accuracies of integrated models are superior to those trained using single modalities [88]. Furthermore, a new generation of trials is currently underway with the aim of marrying neuroimaging, genetics, and behavioral markers of response [124]. Beyond the brain, trials investigating the pharmacogenetics of drug metabolism are showing promise in furthering our understanding of antidepressant response [125•]. Consequently, it is becoming clear that neuroimaging is certainly a promising “tool in the toolkit” and that its integration with other modalities for response prediction is worthy of future investigation.

## Opportunities for Reverse Translation

The discovery of neuroimaging biomarkers of treatment response in humans offers exciting opportunities for reverse translation to animal models, which allow us to uncover detailed mechanisms of antidepressant action and neural substrates of depression. One potential strategy would use fMRI to identify candidate circuits for optogenetic interrogation in rodent models. Opsins could then be used to recapitulate the abnormal functional connectivity features observed in specific circuits in patients and test whether they are sufficient to drive specific depression-related behaviors. One recent example used optogenetic fMRI in rats to show that reward signals arising from the VTA-to-striatum projection are suppressed by top-down mPFC input and that abnormally elevated functional connectivity between the mPFC and striatum is sufficient to produce anhedonia [126]. These findings are consistent with a trend seen in a recent fMRI study showing that greater anhedonia-like symptoms in depressed patients may be predicted by a set of functional connectivity changes involving the mPFC, ventral striatum, and other frontostriatal circuits implicated in reward processing, effort valuation, and motivation [3••].

Future animal studies may employ similar methods to recapitulate the early connectivity changes highlighted in this review. Such efforts could separate connectivity changes which cause recovery from those which merely correlate with recovery, yielding an avenue for future treatment development. Recent work from our lab has highlighted a large number of connectivity features which are correlated with combinations of symptoms in depressed patients [3••]. Combining optogenetics with fMRI may provide a means to determine which of these connectivity changes are sufficient to drive specific depression-related behaviors. Other methods that can be applied directly to humans, including concurrent TMS/fMRI and network-based analyses of lesion studies, are also promising approaches for understanding how dysfunction in specific circuits contributes to specific behaviors [127, 128].

## Conclusions

There is longstanding evidence that brain structure and function is organized into distinct networks of brain connectivity that work in concert to generate complex cognitive patterns and behavior. Research over the past two decades has revealed that abnormal communication within and between these networks underscores the complex psychopathology observed in MDD. Of particular importance to MDD, connectivity of limbic structures, as well as the DMN, SN, CEN, and VMN, give rise to diverse domains of abnormal behavior, including rumination, cognitive control deficits, and anhedonia.

More recent literature provides striking evidence that functional connectivity stemming from networks such as the DMN, SN, CEN, and VMN can classify treatment responders and nonresponders (Fig. 1c). Most encouragingly, divergent sgACC connectivity appears to be predictive of antidepressant response to a variety of interventions, including pharmacotherapy, psychotherapy, rTMS, and DBS. Studies that employ individual-level prediction have identified neuroimaging biomarkers with the ability to retroactively predict treatment response with over 80% accuracy, a clinical benchmark of predictive models. However, methodological variables including patient selection, treatment response criteria, and considerations regarding prediction model overfitting will need to need to be addressed in future studies to ensure that these biomarkers are adequate for prospective prediction of treatment response. Prediction accuracy may also be improved by employing models trained on multimodal data.

Nevertheless, identifying generalizable neuroimaging-based biomarkers of treatment response can diminish the suffering of MDD patients by individualizing treatment and accelerating response rates. Biomarkers related to IBN dysfunction may further our understanding of MDD etiology and improve animal models of MDD, thus driving the development of future interventions for this population marked by limited treatment response.
